# A systematic review on the associations between built environment and mental health among older people

**DOI:** 10.3389/fpubh.2025.1584466

**Published:** 2025-07-02

**Authors:** Yongkang Chen, Lizhen Xu, Xiangfen Cui, Haoran Yang, Yiling Liu, Xin Gao, Jianhong Huang

**Affiliations:** ^1^Faculty of Environmental Science and Engineering, Kunming University of Science and Technology, Kunming, China; ^2^School of Geographic Sciences, East China Normal University, Shanghai, China; ^3^Research Center for Environmental Risk Prevention and Emergency Technology, Yunnan Research Academy of Eco-environmental Science, Kunming, China; ^4^Yunnan Ecological and Environmental Cooperation Office, Kunming, China

**Keywords:** built environment, green space, older people, systematic review, mental health

## Abstract

**Introduction:**

Numerous studies have examined the intricate correlation between community and residential environments, and their impact on health outcomes. However, the influence of built environment (BE) factors on the mental well-being of older individuals varies across different geographical regions.

**Methods:**

This comprehensive systematic review synthesizes research on the association between BE elements and the mental health (MH) of the older adult population. A pool of 2938 studies were reviewed, with 21 studies meeting the inclusion criteria: 17 cross-sectional studies and 4 longitudinal studies.

**Results:**

Most of these investigations were conducted in Asian countries and published within the last five years. The findings reveal that the GDS-15 scale is the most commonly used tool for assessing MH outcomes. We have established a quantifiable evaluation framework for BE elements, addressing the limitations of previous studies that relied on subjective perception measurements. Furthermore, a higher proportion of green spaces, improved facility accessibility, and shorter travel times and distances to destinations are positively linked with better MH of older adults.

**Discussion:**

While these associations are becoming increasingly evident, research on the effects of density, diversity, and design elements in relation to older adults’ MH remains limited and may varied significantly across different regions. Future research should focus on designing quasi-natural experimental studies to enhance our understanding of the convoluted and elaborate relationship between the BE and MH.

## 1 Introduction

The global population is aging at an unprecedented rate, with individuals aged 60 years and older projected to reach 1.4 billion by 2030, accounting for one in six people worldwide (1). While many older adults maintain good overall health, a significant proportion are vulnerable to mental disorders. Data from the Global Burden of Disease (GBD) report estimate that approximately 14% or more of older adults are affected by mental disorders, contributing to 10.6% of disability-adjusted life years in this demographic (2). Depression and anxiety are identified as the most prevalent mental health (MH) challenges. A meta-analysis reveals that the prevalence of depressive symptoms among Chinese reaches is about 20.0% (3). The high prevalence of depressive symptoms in the older adult population significantly poses a substantial financial burden on both individuals and society (4) and elevates national healthcare expenditures (5). For instance, a study in China estimates that the annual medical costs associated with depressive symptoms amount to $42.67 per individual (6). Hence, addressing mental disorders—particularly depression and anxiety—among older adults is a crucial component of promoting mental well-being in alignment with the goals of “healthy ageing” and “active ageing” (7).

Amid the “active health” paradigm, the revitalization of urban public spaces, expansion of green spaces, and advancements in transportation have become key priorities for policymakers, urban planners, and scholars alike. These interventions not only enhance the health of urban population (8, 9) but also promote inclusivity, sustainability, and economic viability (10). In recent years, there has been growing attention to older adults MHs, particularly in identifying root causes and developing comprehensive intervention strategies. The social ecological theory has been instrumental in advancing research in this domain, underscoring that health results from the intricate interplay between environmental factors—encompassing natural, built, and social environments—and individual-based attributes (11). Research consistently shows a strong correlation between the MH of older adults and their socioeconomic status (12–17), social cohesion (18–20), and modes of transportation (21–23). Moreover, urban parks (24–26) and other green spaces (17, 27, 28) are all interconnected in this context. For example, a three-year longitudinal study found that Japanese seniors living in areas with higher connectivity exhibited a significantly lower risk of depression (12–14), emphasizing the importance of the built environment (BE) in fostering mental well-being.

Furthermore, the association between BE elements such as population density, the accessibility of destinations, and depressive symptoms indicates that improved road connectivity and street design can play a pivotal role in enhancing the MH of older female individuals, as supported by recent studies (29, 30). In high-density urban environments, such as Hong Kong, research has shown that changes in building height are negatively related to depression risk among the older adults (31). The ecosystem services provided by green park spaces also promote physical activity among older adults, which, in turn, positively influences their MH (32). Moreover, increasing greenery in the living environment and encouraging social interactions among older adults have been identified as effective strategies for reducing depression and enhancing mental well-being (33–35). These findings underscore the critical need to create inclusive and sustainable urban environments that prioritize the MH and well-being of all citizens.

In the context of “healthy ageing,” medical geographers and environmental epidemiologists have increasingly focused on the complex relationship between the BE and the mental well-being of the older adult population, particularly given the limited scope of their daily activities. However, the intricate and multidimensional nature of both the BE and MH challenges, combined with inconsistencies in how exposure is measured, has led to some incongruence across studies evaluating environmental exposures and health outcomes (36). A systematic review examining the impact of community environments on the physical, cognitive, psychological, and well-being of older adults has found that environmental features play a crucial role in shaping health outcomes in later life. However, specific community environmental factors that are most strongly linked to the MH of older adults remain elusive (37). A systematic review concerning the association between BE factors and depressive mood pinpoints substandard housing quality, scarcity of green spaces, noise, and air pollution as prospective risk factors implicated in the onset of depressive symptoms (38). Although meta-analysis has been conducted to assess the impact of physical community elements on depressive symptoms in older adults, they have often overlooked key attributes of these elements that are closely linked to depression, such as sidewalks quality, traffic safety, and park accessibility. Furthermore, the associations between these environmental factors and MH in older populations remain inadequately understood.

This knowledge gap underscores the need for further research to develop a more nuanced understanding of the factors influencing the mental well-being of older individuals in urban settings. In this context, the present study conducts a comprehensive systematic review of the literature published from 2000 onwards, with the following objectives: (1) to identify prevalent methodologies and instruments used for measuring MH of older adults within this research domain; (2) to delineate key components of the BE that are critical to this field of study, and establish standardized methods for their quantification; (3) elucidate the correlations among diverse facets of the BE and the MH status of the older adult population. This study has unveiled the distinctive vulnerabilities of the older adult population to BE factors through a systematic approach. By establishing a quantifiable evaluation framework for BE elements and an advanced, multi-dimensional evidence-based audit system, we have overcome the fragmented nature observed in early assessments. The research aims to enlighten future studies and offer actionable insights to policymakers and urban planners, enabling them to target interventions that can promote the well-being of the older adult population effectively.

## 2 Methods

### 2.1 Retrieval strategy

This investigation seeks to elucidate the correlation between the BE and the MH of the older adult population. Initially, we examine the complexities of the multidimensional BE and its associated health outcomes. Subsequently, a keyword extraction process is performed, grounded in the various components of the subject. Ultimately, a comprehensive search strategy is developed, integrating multidimensional keywords across three distinct levels: environmental exposures, health outcomes, and the target population.

#### 2.1.1 Built environment

The BE refers to the man-made surroundings designed to accommodate various human activities, including urban settings. In line with contemporary eco-development principles, certain portions of natural environment, such as undeveloped land, are persevered with the BE (8, 9). The BE is a multifaceted construct, with different scholars focusing on various elements that contribute to its composition. The “5D” model—Density, Diversity of land use, Design, Destination accessibility, Distance to transit— is commonly used to describe the urban BE (39). Furthermore, studies have increasingly integrated green parks and urban water bodies as essential components for urban design, contributing to ecological character of urban environments.

Drawing from existing research literature (12–14, 40), this study focuses on six key elements of the BE: (I) **Density**: Factors such as building density, population density, and street density; (II) **Design**: Aspects related to the exterior style of buildings, building materials, and the planning of public spaces; (III) **Diversity**: The range of functions within buildings and communities, as well as the availability of cultural outreach services; (IV) **Facility accessibility**: The ease of access to critical facilities such as parks, pharmacies, food markets, schools, and healthcare services within buildings and neighborhoods; (V) **Road distance**: The connectivity of streets and roads, as well as the proximity of dwellings to key facilities. Additionally, green space―encompassing both natural and man-made landscapes including parks, courtyards, community gardens, and forests―are also considered.

#### 2.1.2 Mental health

MH refers to a state of psychological homeostasis, where an individual experience a sense of contentment and is able to effectively manages life’s challenges. According to the World Health Organization’s (WHO) *World Mental Health Report*, MH is defined as an enabling state that allows individuals to realize their potential, excel in learning and work, and contribute meaningfully to their communities (41). As individuals age, a natural decline in MH and cognitive functioning often occurs, which may manifest as reduced cognitive ability, memory loss, and diminished intellectual capacity. Older adults are especially vulnerable to negative emotional states, including loneliness, depression, and boredom, and are at higher risk of developing psychiatric disorders such as intellectual disability, depression, and delusional disorders (8, 9, 42). These conditions serve as significant and prevalent indicators for assessing the MH status of the older adult population, thereby providing a comprehensive representation of their overall MH level.

#### 2.1.3 Older people

The term ‘old age’ has traditionally been defined as beginning at 65 years and older. Within this classification, subgroups are often distinguished as “early old age” (ages 65 to 74 years) and “late old age” (ages 75 years and beyond) (43). Notably, the WHO defines the older adult population as those aged 60 years and older, while nutritional studies typically use a threshold of 55 years. In this study, we classify the older adult population based on two criteria: (1) studies involving populations aged 60 years or older, or (2) studies where the average age of the population exceeds 65 years, even if a threshold of 55 years is applied.

#### 2.1.4 Retrieval strategy

To conduct a systematic evaluation and meta-analysis, we adhered to the Preferred Reporting Items for Systematic Reviews and Meta-Analyses (PRISMA) guidelines (44). The search covered publications from 1st January 2000 to the present. This study systematically examines terms related to the built and urban environment, residential communities, green spaces, older adults, and their mental and psychological well-being, utilizing a comprehensive set of 72 keyword combinations (Supplementary Table S1). Focusing on the environmental factors of “built environment” and “green space,” a range of synonyms was employed to ensure a comprehensive coverage of relevant research. This multi-dimensional and multi-synonym approach enables the retrieval of a wide array of literature, maximizing both the comprehensiveness and accuracy of the literature review. It is crucial to include multiple synonyms for terms such as “older adults” and “mental health” to ensure that no important research on the mental health of older adults is overlooked. This ensures that all relevant literature on the topic is retrieved, providing a comprehensive understanding of the subject matter.

The most frequently retrieved keyword combinations in the *Web of Science Core Collection* were: “urban environment, older people, mental health”; “community environment, older people, mental health”; “community environment, older adults, mental health.” In the PubMed database, the leading combinations included: “community environment, older adults, mental health”; “community environment, older adults, mental health”; “community environment, older people, emotional well-being.” A detailed breakdown of the number of articles retrieved for each combination is provided in (Supplementary Table S1). In total, we retrieved 2,938 articles, representing a comprehensive examination of the relationships between BE, urban settings, community environments, residential areas, green spaces, and the mental and psychological well-being of older adults.

### 2.2 Inclusion and exclusion criteria

The inclusion criteria within this study were designed to accurately capture the intricate and subtle impacts of characteristics inherent in the BE on the MH of the older adult population. First, the mean age of the entire sample or any analyzed sub-sample must be 65 years or older, aligning with the focus of the study. Second, selected studies must present evidence linking environmental exposure indicators—such as density, design, diversity, facility accessibility, road distance, and green space—to MH outcomes in older adults. These factors are essential for understanding the psychological implications of the BE. Third, the health outcomes investigated must include overall MH or common psychological disorders, such as depression and anxiety, which reflect the mental state of older adults in relation to their environment. Fourth, non-journal sources, such as books, case reports, conference proceedings, government publications, and technical reports, were excluded to ensure the inclusion of peer-reviewed, high-quality research. Finally, only articles published in English were considered to ensure global accessibility and consistency. By adhering to these criteria, this study aims to provide a thorough and comprehensive assessment of the association between the BE and the MH of the older adult population.

### 2.3 Literature selection

**Figure 1:**
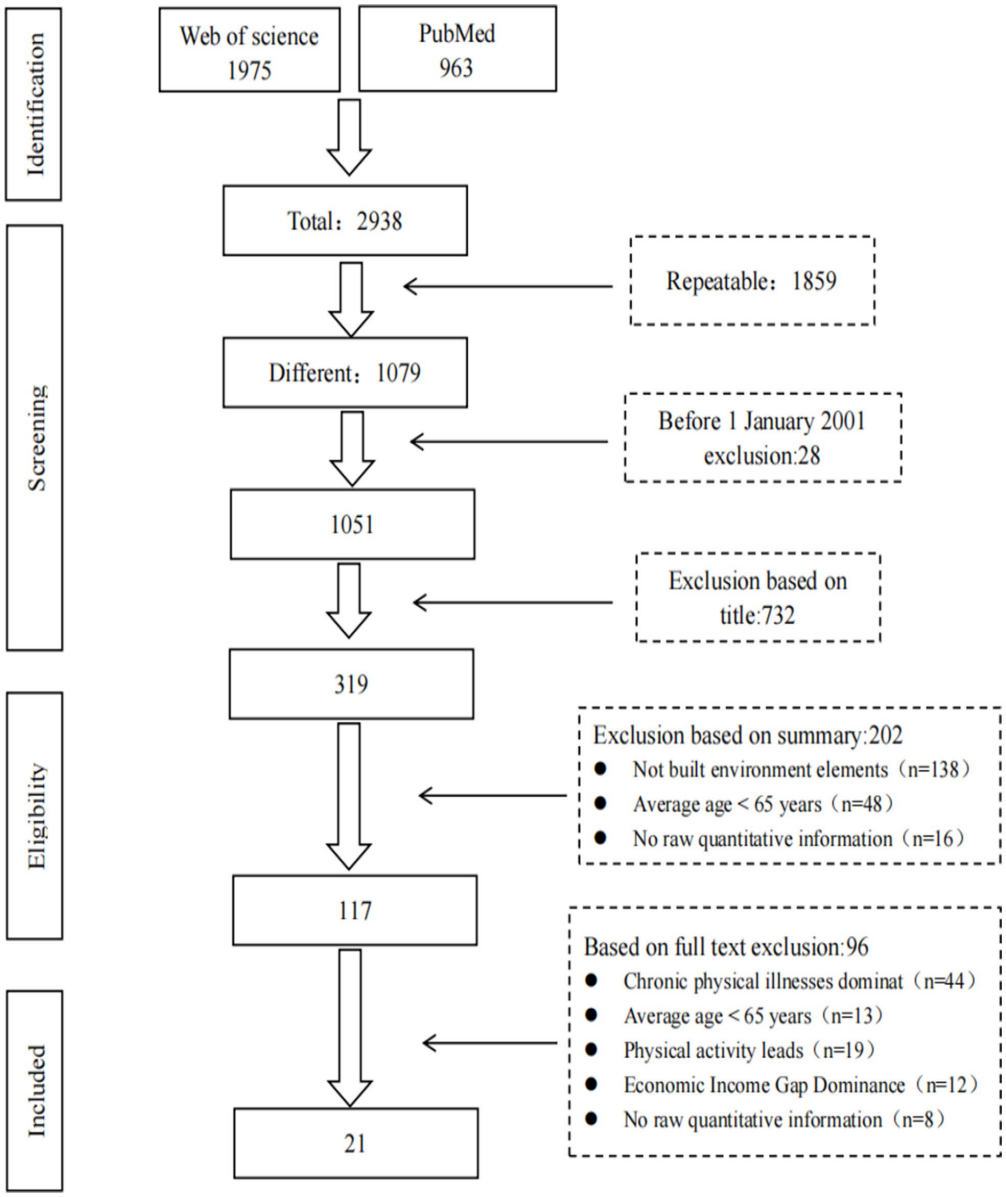
Flowchart of literature selection.

The literature selection process, depicted in Figure 1, resulted in the final inclusion of 21 articles for analysis. Initially, 2,938 articles were retrieved. Using the “*Find Duplicates*” tool in Endnote X9, 1859 duplicate records were removed, leaving 1,079 unique literature. A subsequent title and abstract screening eliminated 934 irrelevant records, reducing the pool to 117 articles for full-text review. During the full-text review process, 96 articles were excluded for not meeting the stipulated inclusion criteria. Common reasons for exclusion including insufficient focus on older adult, lack of analysis of BE factors, or absence of primary quantitative data. Ultimately, 21 articles were deemed suitable for data extraction and analysis, ensuring the study’s robustness.

To enhance comprehensiveness, three key review articles (15, 36, 38) were scrutinized, including their bibliographies. This additional effort identified one overlooked article (45) that offered valuable insights into longitudinal associations between green space changes and MH. However, it was excluded from the initial analysis due to its lack of specific focus on the older adults.

### 2.4 Variable information extraction

To achieve study objectives and elucidate the associations between older adults MH and various dimensions of the BE, we conducted a meticulous data extraction process. This process involved gathering key information from each study, including the first author, publication year, study design, and location. We also collected detailed participant data, such as sample size and male-to-female ratio. The study design was comprehensively documented, covering MH outcome, BE domains, significant variables, and statistical methods (Tables 1, 2). The extracted variables are carefully categorized into distinct groups. For instance, indicators of environmental exposure are segmented based on the dimensions of BE elements—such as density, design, diversity, facility accessibility, road distance, and green spaces. Health outcome indicators are segmented according to overall MH and prevalent mental disorders, specifically depression and anxiety. For our research conclusions, we have organized and summarized the findings based on the direction (positive or negative correlation, or no correlation) of the association found between BE elements and MH in the older adult population.

**Table 1:** General characteristics of the studies included in this review.

No.	Reference	Country	Sample size	Male	Female	Healthy	Unhealthy	Measurement	Sample method	Research method	Research design type
1	Arias et al. (46)	Spain	4,729	3,659	1,070	2,605	2,124	GHQ-12	Multistage sampling	Logistic regression model	Cross sectional
2	Chen et al. (11)	China	938	391	547	561	377	GDS-15	Multistage sampling	Logistic regression model	Cross sectional
3	Hanaza et al. (2)	Japan	24,141	10,614	11,235	21,849	2,292	GDS-15	JAGES	Logistic regression model	Longitudinal
4	Domènech-Abella et al. (71)	Spain	5,912	2,496	3,416	4,848	1,064	CIDI 3.0	Stratified sampling	Logistic regression model	Cross sectional
5	Guo et al. (48)	Hong Kong	1,553	1,200	353	843	710	SF-12	Stratified sampling	Structural equation modeling	Longitudinal
6	Ho et al. (72)	Hong Kong	3,930	1,965	1,965	3,566	364	GDS-15	Random sampling	Logistic regression model	Cross sectional
7	Koohsari et al. (29)	Japan	328	124	204	244	84	GDS-15	Random sampling	Hierarchical regression models	Cross sectional
8	Lam et al. (49)	Hong Kong	347	79	268	257	90	GDS-15	Random sampling	Structural equation modeling	Cross sectional
9	Lee et al. (33)	Korea	11,408	4,922	6,486	8,214	3,194	GDS-15	CHS	Logistic regression model	Cross sectional
10	Liang et al. (55)	Sichuan	515	194	321	155	360	WHO-5	Random sampling	Logistic regression model	Cross sectional
11	Lu et al. (50)	Hong Kong	1,277	269	1,008	536	741	SWEMWBS	snowball sampling	Structural equation modeling	Cross sectional
12	Liu et al. (59)	Hong Kong	2,081	916	1,165	793	1,342	GDS-15	Random sampling	Structural equation modeling	Cross sectional
13	Noordzij et al. (45)	United States	4,118	1,976	2,142	1,812	2,306	CSD -11	NSHAP	Mixed effects linear regression model	Longitudinal
14	Vegaraju and Amiri (34)	United States	48,623	21,881	26,742	18,607	24,373	HRQOL-4	BRFSS	Logistic regression model	Longitudinal
15	Yue et al. (8, 9)	Dalian	879	406	473	/	/	WAVE 1	Random sampling	Hierarchical regression models	Cross sectional
16	Zhang et al. (25, 26)	Guangdong	932	405	527	/	/	SF-36	Multistage sampling	Multilevel linear model	Cross sectional
17	Wu et al. (53)	Beijing	757	321	436	679	78	GDS-15	Random sampling	Multilevel linear model	Cross sectional
18	Helbich et al. (52)	Beijing	1,190	476	714	/	/	GDS-15	Stratified sampling	Hierarchical regression models	Cross sectional
19	Wang et al. (35, 54)	Shanghai	7,512	3,490	4,022	/	/	GDS30	Random sampling	Mixed effects linear regression model	Cross sectional
20	Wang et al. (67)	China	993	403	590	/	/	HDS	Random sampling	Boruta algorithm	Cross sectional
21	Nishida et al. (66)	Japan	13,1871	63,430	68,441	/	/	GDS-15	JAGES	Logistic regression model	Cross sectional

Note: “/” indicates that the corresponding article does not have a quantitative description. GHQ, General Health Questionnaire; GDS, Geriatric Depression Scale; CIDI 3.0, Composite International Diagnostic Interview; SF (Short Form) is designed to assess the health-related quality of life; SWEMWBS, the seven-item short Warwick–Edinburgh Mental Well-being Scale; CDS-11, the 11-item Center for Epidemiological Studies – Depression Scale; WAVE 1, The WAVE survey is a longitudinal study of aging; HDS, Hamilton Depression Scale; JAGES, Japan Gerontological Evaluation Study; CHS, Community Health Survey data from the Korea Centers for Disease Control and Prevention; NSHAP, NSHAP is a longitudinal, nationally representative study the US; BRFSS, Behavioral Risk Factor Surveillance System.

**Table 2:** Characteristics of built environment elements included in the studies of this review.

No.	Reference	Built environment elements						Independent variables	Significant BE factor
		Density	Design	Destination	Diversity	Distance	Greenspace		
1	Arias-Fernández et al. (46)			✓			✓	A, C, E, G, M, I. air pollution, green spaces, smoking, alcohol consumption	Green space and air pollution
2	Chen et al. (11)	✓	✓					A, C, E, G, M, I. building density, sleep duration, house layout	Building density
3	Hanaza et al. (2)	✓	✓	✓				A, C, E, G, M, I. population density, intersection density, street connectivity	Street connectivity
4	Domènech-Abella et al. (71)		✓	✓				A, C, E, G, M, I. facility accessibility, road network density	Facility accessibility
5	Guo et al. (48)	✓		✓			✓	A, C, E, G, M, I. housing type, street connectivity, accessibility, green space	Destination distance
6	Ho et al. (72)	✓						A, C, E, G, M, I. building density, building height	Building density
7	Koohsari et al. (29)	✓	✓					A, C, E, G, M, I. population density, destination accessibility, street density	Street density
8	Lam et al. (49)			✓			✓	A, C, E, G, M, I. land use mix, connectivity, parkland	Facility accessibility, green space
9	Lee and Lee (33)						✓	A, C, E, G, M, I. physical activity, smoking, alcohol consumption, green space	Green space
10	Liang et al. (55)			✓		✓		A, C, E, G, M, I. transport connectivity, accessibility to destinations	Facility accessibility
11	Lu et al. (50)			✓				A, C, E, G, M, I. economic status, facility accessibility, walking distance	Facility accessibility
12	Liu et al. (59)			✓			✓	A, C, E, G, M, I. cognition, facility accessibility, green space	Facility accessibility, green space
13	Noordzij et al. (45)			✓		✓		A, C, E, G, M, I. smoking, physical activity, air pollution	Facility accessibility, green space
14	Vegaraju and Amiri (34)						✓	A, C, E, G, M, I. green space	Green space
15	Yue et al. (8, 9)						✓	A, C, E, G, M, I. green space	Green space
16	Zhang et al. (25, 26)	✓		✓		✓		A, C, E, G, M, I. accessibility to facilities, building density, distance to bus stops	Density, facility accessibility, distance to destination
17	Wu Zhifeng and Yin (53)						✓	A, C, E, G, M, I. air pollution, blue-green space	Green space
18	Helbich et al. (52)						✓	A, C, E, G, M, I. blue space, green space	Green space
19	Wang et al. (35, 54)	✓					✓	A, C, E, G, M, I. smoking, alcohol consumption, residential greenery	Green space, density
20	Wang et al. (67)		✓				✓	A, C, E, G, M, I. residential floor, green space	Green space, density
21	Nishida et al. (66)			✓		✓		A, C, E, G, M, I. Destination distance	Destination distance

The symbol “✓“is used here to indicate that the corresponding BE (Built Environment) domain was studied. In order to reduce repetition, use abbreviations for the following variables: A, Age; C, Chronic diseases; E, Education level; G, Gender; M, Marital status; I, Household income.

## 3 Results

### 3.1 General characteristics of the research

The study included older populations from diverse countries, with the majority of participants hailing from Asia. China contributed the largest proportion, accounting for 61.9% of the participants (*n* = 13), followed by studies from South Korea (4.8%, *n* = 1) (33), Japan (14.3%, *n* = 3) (12–14, 29), Spain (9.5%, *n* = 2) (46, 47) and the United States (9.5%, *n* = 2) (30, 34). These studies offer a cross-cultural perspective, including settings such as Hong Kong (5 studies) (31, 48–51), Beijing (2 studies) (52, 53), and various cities in China (54), Dalian (8, 9), Guangzhou (25, 26), and a county in Sichuan (55) as well as international sites (Spain, the United States, South Korea, and Japan). This diversity allows for a comprehensive and nuanced analysis of the various factors that impinge upon the MH of the geriatric cohort within disparate contexts.

Most studies employed cross-sectional studies (80.9%, *n* = 17), with longitudinal studies making up 19.1% (*n* = 4). For publication year, a significant proportion (80.9%, *n* = 17) were published after 2020, indicating a growing recognition of the role of BE in influencing the MH of older adults. Four studies (19.1%, *n* = 4) were published in 2019, with one study from 2017.

Regarding sampling method, 42.9% (*n* = 9) of studies used random sampling, while 23.8% (*n* = 5) utilized country-level health survey data. Multi-stage and stratified sampling methods were used in in three studies (14.3%) of studies each, and snowball sampling was used in just one study (4.8%). Sample sizes varied widely, from 328 to 131,871 participants (Table 1). National health surveys contributed the largest samples, with over 10,000 participants in three studies. Most studies with random sampling have sample sizes under 1,000, while studies using multi-stage or stratified sampling had sample between 1,000 and 5,000. Regarding gender distribution, nine studies reported nearly equal male-to-female ratios, while the remaining 10 studies had a significant preponderance of females. A detailed account of the presence and absence of MH concerns within the investigated population is furnished in Table 2, presenting significant data conducive to a more profound exploration of the correlation between the BE and MH among the older adults.

### 3.2 Measurement tools for MH outcomes and their geographic variation

**Figure 2:**
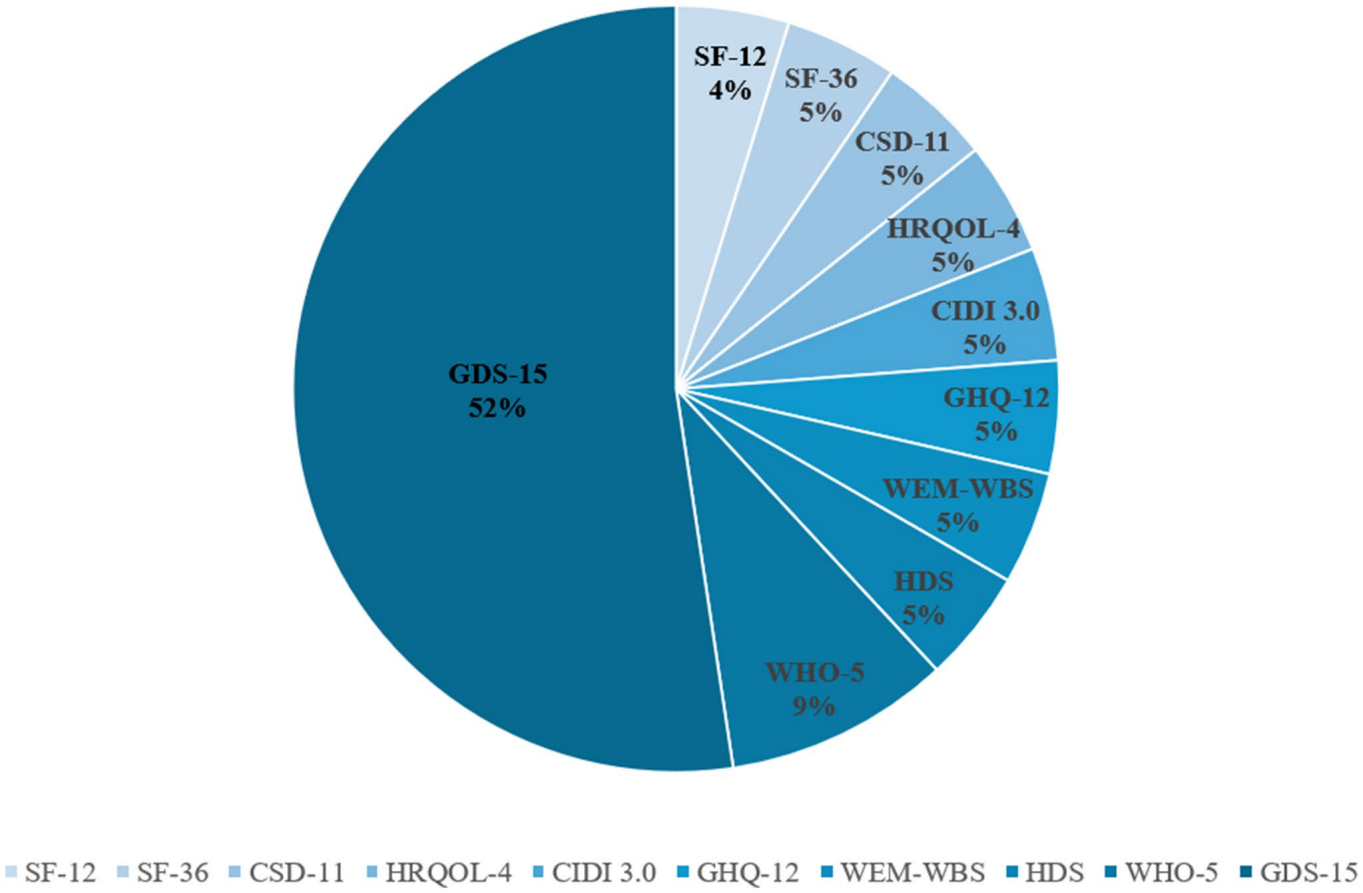
Methods of measuring mental health.

In the 19 studies reviewed, a diverse array of nine distinct instruments were employed to assess MH outcomes in older adults (Figure 2). Among these, the *15-item Geriatric Depression Scale* (GDS-15) stands out as a specialized tool designed especially for the older adults. Comprising 15 items, the GDS-15 captures key depressive symptoms commonly observed in older adults, including mood disturbances, reduced activity levels, irritability, social withdrawal, distressing cognitive processes, and adverse appraisals of the past, present, and future experiences. This scale provides a standardized and comprehensive means for evaluating depression in this demographic. Another prominent instrument is the *11-item Centre for Epidemiological Studies Depression Scale* (CES-D11), which evaluates six dimensions of depression: mood disturbance, guilt, helplessness, psychomotor retardation, appetite loss, and sleep disturbances. The CES-D11 offers a broad and detailed assessment of depressive symptoms.

The *12-item health-Related Quality of Life Scale* (SF-12), a concise version of the SF-36 Scale, is frequently utilized to assess quality of life in both the general population and individuals with chronic conditions. Additionally, the *5-item WHO Well-being Index* (WHO-5) is one of the most widely used tools for measuring subjective MH, offering a reliable assessment of overall well-being. Other instruments include in the reviewed studies are the *4-item health-related quality of life scale (HRQOL-4)*, which assesses physical, mental, and social aspects of health-related quality of life, and the *International Diagnostic Interview* (CIDI), a structured diagnostic tool primarily used in epidemiological studies to assess of MH disorders. The GHQ-12, a self-report scale consisting of 12 items, assessed depression, anxiety, social functioning, and overall health status. The *Hamilton Depression Scale* (HDS) is the most widely used scale in clinical evaluation of depression. Lastly, the *Warwick-Edinburgh Positive Mental Health Scale* (WEM-WBS) integrates three key dimensions of MH—positive emotions, positive mental functioning, and relationship satisfaction—across its 14 items.

In our review, over half of the studies (*n* = 11, 52.4%) employed the GDS-15 to assess the MH of older adults, with a strong focus on studies conducted in China, South Korea, and Japan. Notably, two studies from China used data from the WHO’s WAVE-1 survey, which assesses the mental well-being of older populations. Additionally, two studies used brief MH questionnaires: one conducted in Hong Kong utilized the SF-12, while another study from Guangzhou employed the SF-36. Five other distinct MH measures were used in individual study. These included the CSD-11, which was used in a study from Washington State, United States, that explored the connection between the BE and MH by leveraging data sourced from the National Health Database. In Span, three different tools were used: the *12-item General Health Questionnaire* (GHQ-12) assessed the MH of older people in relation to the physical community environment, a revised version of (CIDI 3.0) was used in another study, and the *Warwick-Edinburgh Mental Health Scale* (WEM-WBS) was employed to explore the mediating role of social capital and community structure.

In the realm of psychological health assessments, notable disparities exist in the efficacy of current MH measurement tools when it comes to capturing the psychological well-being of older adults. These disparities primarily stem from distinct design objectives, symptom dimensions, and cultural applicability. Diagnostic specificity instruments, such as the CIDI, are structured clinical diagnostic tools with a modular design that facilitates a systematic screening for positive symptoms like hallucinations and delusions (46). However, their intricate operational procedures, which often take an average of 45 min, can contribute to response fatigue and introduce a tendency for false-negative biases in epidemiological studies involving older adult populations. On the other hand, broad-spectrum screening tools like the GHQ-12 and SF-12 encompass dimensions such as anxiety and social function (48). Yet, they exhibit a lack of sensitivity toward specific symptoms associated with MH, like thought disorder and affective flattening. This can lead to confusion with symptoms of comorbidities commonly experienced by the older adults, such as dementia. Among the available tools, the GDS-15 stands out due to its optimization with age-specific items (52). These include considerations like “loss of interest in the past” and “reduced activity,” effectively capturing psychological distress stemming from negative symptoms. What further enhances its reliability is its concise nature—being completed in just 5 min—and its use of language that is easily understood by older adults. This combination ensures that the tool not only efficiently assesses psychological health but also fosters trust and openness in the responses provided by the older adult population.

Cultural adaptability differences further enhance the differentiation of tool validity: The preference for GDS-15 in Asian studies stems from its targeted capture of the mechanism of geriatric depression —older adults patients with MH often present with “hidden negative symptoms” such as emotional withdrawal and lack of motivation, and the item structure of GDS-15 (such as “feeling empty in life” and “avoiding social activities”) precisely aligns with such symptoms. In contrast, while European and American tools (such as CIDI, SF-12) cover a broader spectrum of psychopathology, their cross-cultural validity is significantly reduced in Asian older adult populations. Based on the above MH measuring tools, the GDS-15 has predominantly been employed in studies conducted in Asia, while European and American studies have embraced a more diverse array of instruments, thereby highlighting distinct regional preferences in measuring mental health outcomes. This instrument, specifically designed for the older adults, has blossomed into the most efficient tool in assessing the psychological ramifications associated with mental health in the older adult population.

### 3.3 BE elements and their measurement

**Figure 3:**
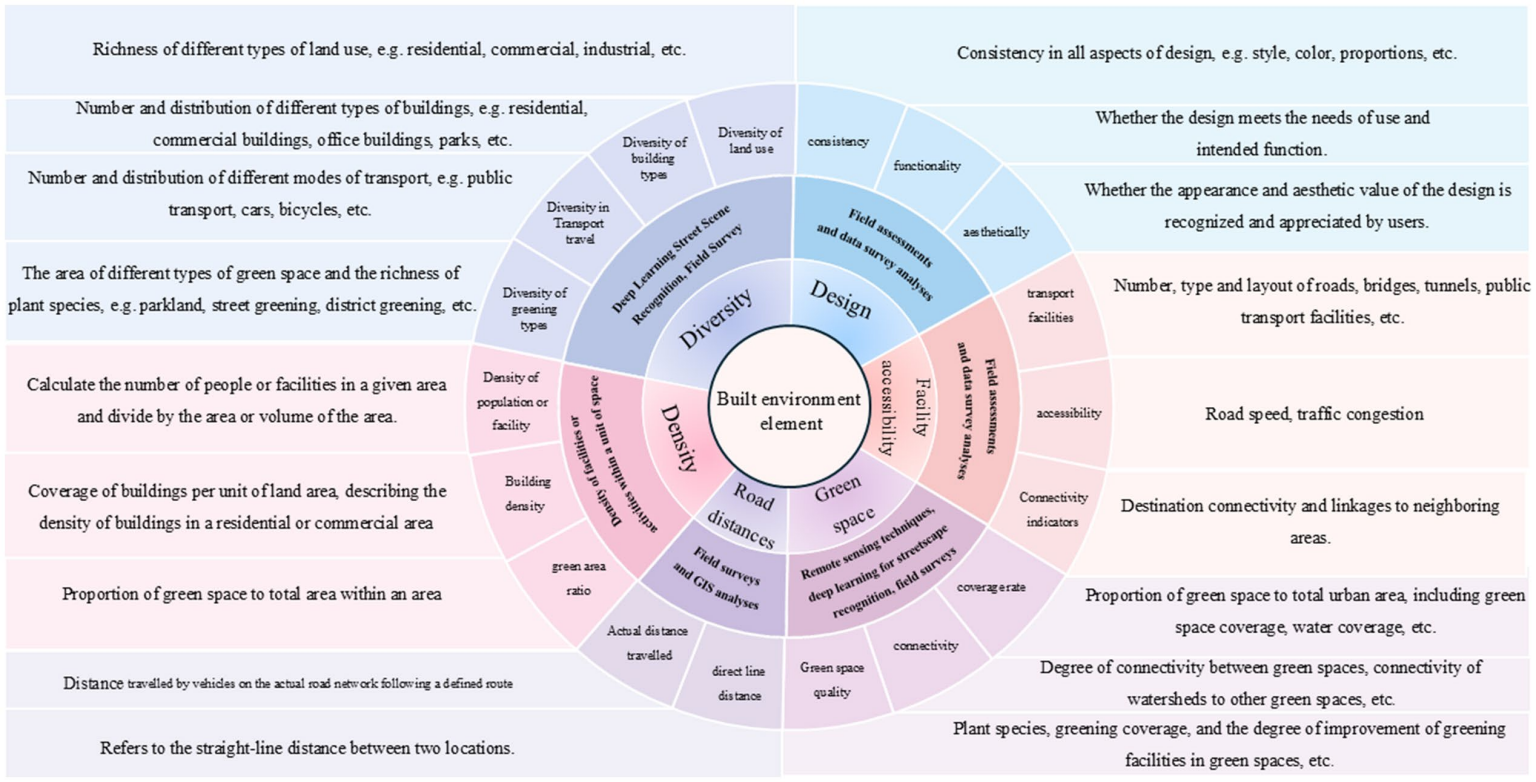
BE elements and their measurements.

As illustrated in Figure 3, our analysis systematically summarizes the six dimensions of the BE. To gain a comprehensive understanding of the BE and its impact, we employ a combination of field observations and measurements to derive design-related data indicators. These indicators are obtained through various methods, including questionnaires and statistical analyses. Additionally, we assess the functionality of the design to evaluate its effectiveness and identify potential areas for improvements. This comprehensive approach enables a nuanced and detailed understanding of the BE’s multiple dimensions. BE measurements are widely supported by Geographic Information System techniques, combined with remote sensing technology and on-site surveys.

### 3.4 Associations between elements of the BE and MH

As presented in Table 2, green space emerges as the primary BE element influencing the MH of older individuals. Notably, 52.4% (*n* = 11) of the studies reviewed highlight a significant association between exposure to green space and mental well-being (34). Seven of these studies focused specifically on the importance of green spaces, while eight studies (38.1%, *n* = 8) underscore the critical role of accessible facilities, particularly pavement width and the availability of street connectivity (12–14). In addition, four studies (19%, *n* = 4) examined the correlation between building density and MH, with research findings suggesting that individuals residing high-density areas exhibit an elevated susceptibility to MH issues (31). Moreover, a subset of studies (19%, *n* = 4) analyzed the influence of proximity to destinations, finding that services accessible within a 300-meter radius significantly positive association with MH (50). Notably, only one study explored the impact of housing design on MH, indicating a gap in the literature regarding the effects of diverse community functions and cultural amenities. These findings underscore the multifaceted role that BE elements play in shaping the MH of older individuals.

**Green space**. Green space has garnered significant attention in studies investigating the association between the urban BE and the MH of older adults, highlighting its crucial role in promoting mental well-being. Specifically, 10 studies (52.4%, *n* = 11) have explored the impact of exposure to blue and green spaces on older adults’ MH, with seven focusing exclusively on green spaces. The remaining three studies examined the synergistic effects of green spaces linking with the accessibility and density of facilities. The importance of urban green spaces is consistently emphasized across multiple studies (46). For example, a study conducted by Vegaraju and Amiri (34) in urban Washington State found that older adults living near blue-green spaces, particularly those with tree canopies and forests, reported better self-rated health. Similarly, (33) found a positive correlation between proximity to green spaces and better MH in South Korea. Another study shows positive effects of higher proportion of green space on MH, specifically dementia and depression (56). Additional studies from China also found significant benefits of green spaces exposure, park vegetation coverage and proportion of residential greenery, particularly in reducing depressive symptoms among seniors (8, 9, 57). We recommend that expanding urban and community green spaces may serve as a preventive and intervention measure to mitigate depressive symptoms in this demographic.

**Facility Accessibility**. Several studies (38.1%, *n* = 8) emphasize the significance of facility accessibility in the community, particularly focusing on pavement width and the availability of street seating. A study by Domènech-Abella et al. (47), which examined a cohort of non-institutionalized older individuals from Finland, Poland, and Spain, found that enhancing community infrastructure such as walkability and facility accessibility can mitigate loneliness and alleviate depressive symptoms in older adults. Further studies by Koohsari et al. (29) and Lam et al. (49) corroborated these findings, highlighting that environments conducive to walking, with high population densities and access to local amenities, can significantly improve the MH of older adults, particularly older women. For instance, increasing walkability and providing more neighborhood parks were found to have a direct positive impact on the mental well-being of seniors in Hong Kong and Singapore.

**Building Density**. A smaller subset of studies (19%, *n* = 4) has explored the relationship between building density and older people MH. High-density cities present a unique context for understanding this relationship. In Hong Kong, China, a higher proportion of residential areas and taller buildings were found to correlate with poorer MH outcomes for older adults. Studies by Ho et al. (31) and Chen et al. (12–14) confirmed this negative correlation between building height and mental well-being. Conversely, a study from Japan conducted by Chen et al. (12–14) revealed that older individuals living in areas with higher street intersection density and better street connectivity reported better MH than those in less connected areas.

**Proximity to Destinations**. Four studies (23.8%, *n* = 5) examined the relationship between older people MH and the proximity of various destinations. One such study specifically looked at the accessibility of parks, convenience stores, supermarkets, and farmers’ markets, finding that services within 300-meter radius of a resident’s home were crucial for mental well-being (48). Another study conducted in rural areas revealed that distance from the town center or coach terminal negatively affected the MH of older individuals (55). These studies highlight the importance of local services and amenities in improving the MH of senior citizens.

**Housing design**. Only one study focusses on housing design as a significant factor influencing older adults’ mental well-being, suggesting that suggesting that more attention should be given to housing design in future studies (11). No studies directly addressed the diversity of BE elements or the impact of cultural services in the community, which could offer valuable insights into MH outcomes for older adults. These two areas are identified as important topics for future research.

### 3.5 Potential pathways linking BE to MH

The BE, as a crucial external factor influencing the health of residents, is intricately linked with the mental well-being of the older adults through its six core components: density, diversity, design, destination accessibility, street distance, and green spaces. In terms of density, a moderate level promotes social interactions and fortifies the social support network of the older adults, ultimately reducing feelings of loneliness. Yet, an excessively high density may create a sense of crowding and noise, potentially augmenting psychological stress (11). Regarding diversity, the richness of land use, building types, and transportation modes offers a plethora of choices to the older adults for socializing, leisure, and activities. This diversity caters to their diverse needs and fosters a sense of psychological well-being. Conversely, a monotonous BE can lead to a mundane lifestyle and increase the risk of depression. Furthermore, design elements play a pivotal role. An environmentally friendly design that combines functionality and esthetics creates an environment that is both conducive and appealing (29). For instance, a well-planned layout of building spaces and the provision of age-appropriate facilities can significantly enhance the older adult’s satisfaction and sense of belonging to their surroundings. Conversely, an environment void of humanized design may lead to difficulties in mobility and psychological discomfort.

The accessibility of destinations is paramount in determining the social engagement level of the older adults (45). If equipped with convenient transportation facilities and efficient travel routes, the older adults can readily access healthcare, socialize, and visit leisure spots, diminishing their feelings of isolation. In contrast, limited accessibility constrains their range of activities and may exacerbate psychological barriers. Regarding the elements of street distance, excessively long driving distances and time constraints can diminish the older adult’s desire to travel and hinder social interactions. However, short and seamless street distances foster their daily activity frequency and uphold their MH (22). Moreover, sprawling green areas not only provide fresh air and captivating landscapes to alleviate the mental fatigue of the older adults but also promote outdoor activities and foster connections with nature through well-connected ecological spaces. This positive impact eases moods and alleviates anxiety and depression.

In conclusion, the six core elements of the BE exert profound impacts on the MH of the older adults, influencing them directly and indirectly through various facets such as social interaction, activity participation, and environmental perception. In urban planning and environmental design, it is of utmost importance to fully consider these elements and create a BE that caters to the lives of the older adults. Such an endeavor holds great significance for promoting the MH of the aging population and enhancing the quality of life in their later years.

## 4 Discussion

### 4.1 BE elements and MH

The nexus between the BE and the MH of the geriatric population remains an area that is inadequately investigated. This review analyzed 21 studies that investigating this interplay, noting a significant increase in research over the past 5 years. This trend highlights a growing recognition of the BE’s pivotal role in fostering MH among seniors. In-depth research on the older adults highlights the multi-layered and dynamic effects of the environment on their MH. This research emphasizes the significance of considering factors such as life stage experiences and changes in social roles that influence their interactions with the BE (73). In contrast, studies on other populations often prioritize theories like social support theory and stress coping theory, focusing on the relationship between environmental stressors and individual coping strategies (74). Research efforts in this area have predominantly been conducted in Asia, particularly in China, reflecting the region’s rapidly growing older adult population. However, this geographic bias underscores the importance of conducting border studies across diverse regions to inform the development of sustainable and inclusive healthcare systems worldwide. To better understand and address these dynamic interactions, future investigations should adopt a healthy aging perspective, considering the unique experiences and challenges faced by older adults in different contexts. This approach will contribute to a more comprehensive understanding of the complex relationship between the BE and the MH of the older adults, ultimately leading to improved healthcare interventions and policies that promote their well-being.

Regional heterogeneity is manifest in the correlation between the BE and MH. Studies consistently show that increased green spaces, enchanted facility accessibility, and closer proximity to destination are linked to improved MH. For example, amidst the contemplation of the biophilia hypothesis and its intricate interplay with psychological research, an implicit affirmation arises from the vital function played by urban green spaces. These green havens nurture our innate biophilic tendencies, thereby indirectly substantiating the positive influence of biophilic design in mitigating psychological stress and fostering emotional stability among the older adults (75). As we delve into the health implications of various street-level green interventions, the findings emerge with a clear correlation: the greener one’s local environment, the higher their level of well-being and the lower their perceived stress levels. These street-level green interventions possess a positive impact on individuals’ sense of well-being and stress associated with urban environments (76). A Nature-styled investigation into the correlation between psychological distress and green spaces in Australian adults has revealed that individuals residing in neighborhoods abundant in foliage are at a significantly reduced risk of developing psychological distress in comparison to those residing in the least verdant areas (58). Middle-aged and senior citizens residing in green neighborhoods exhibit improved MH, attributed not solely to the promotion of physical activity (59), but rather to the heightened social cohesion in green spaces and the mitigation of air pollution. Green spaces can alleviate stress, elevate overall mood, and enable individuals to engage with their environment through all five senses, thereby enhancing their well-being (60). A study conducted in the north-western Italian city of Turin has found that easy access to public transportation, along with a dense urban structure, diminishes the likelihood of depression, particularly for women and older adults, by providing more opportunities for mobility and social engagement (61). However, research on density is limited, with only five studies addressing this aspect in high-density urban areas such as Hong Kong, Guangzhou, and Shanghai and Tokyo. These studies suggested that higher building densities and intricate street networks may elevate the risk of MH issues among older adults. Notably, the absence of a universal definition for high density and its implications for MH warrants further investigation. Research on the diversity and design of BE elements remains scarce, possibly due to a perceived lesser impact on daily life compared to other factors. Nevertheless, our comprehensive and systematic analysis corroborates that the BE exerts a substantial and significant role in sustaining the mental well-being of the older adult population. Future research should prioritize tailoring urban planning and design to address the unique needs of this demographic.

Contrary to prevailing assumptions, some studies reported negligible associations between BE elements and MH among older adult populations. For instance, a Canadian national health survey (62) found no significant association between physical activity and green space use among seniors. Similarly, research in Germany (63) observed no robust connection between proximity to urban green spaces MH among population aged over 50 years. Some studies conducted in Europe and the United States also reported that rising near green space does not significantly correlate with self-reported psychiatric symptoms or formal mental illness diagnosis (64). A Dutch study using fixed-effects analysis showed no notable shifts in older adults’ mental well-being associated with alterations in green space exposure (45). In a comprehensive survey conducted in Hong Kong, involving 1,553 older individuals, the exploration was conducted regarding the perception of the influence of the BE and the sense of community on mental health. The findings revealed that living density within 300- and 500-meter buffer zones did not exhibit a significant association with mental health. Furthermore, the proximity of shopping and community services was found to have neither a direct nor an indirect influence on mental health or subjective well-being (48). The insignificant effect of green spaces on people’s health may be attributed to the study population’s unique characteristics. Older adults, often constrained by physical limitations and chronic illnesses, tend to be less inclined to travel long distances to utilize green spaces. Consequently, they are unable to actively leverage green spaces as a means to promote their health (65).

A comprehensive study conducted in the city of Dalian, China, investigated the relationship between the street network and the MH of older people. Utilizing data from 879 respondents aged 60 years and above, it was observed that the connectivity of pedestrian-friendly streets and carriageways at the street level did not have a significant association with the mental well-being of the older adult population (8, 9). Additionally, the study explored the correlation between the proximity of a crucial amenity in Japanese communities, namely elementary schools, and depression in older adults. Surprisingly, the findings indicated that the proximity of elementary schools was not notably linked to depression in older men (66). A study conducted in a hospital in China, examining the relationship between individual mental health and environmental risk factors through clinical data and outpatient surveys, revealed that the elevation of living floors had no discernible impact on mental well-being (67). For older adults, higher intersection densities may pose a hindrance to active living and mental health due to inadequate road safety during crossings (68). Moderate levels of street connectivity can be beneficial for older individuals as pedestrian-friendly designs encourage mobility and consequently enhance their mental health (48). The reason for the absence of an association in the study may be attributed to older individuals’ predominantly neighborhood-based daily activities (69), which often involve short trips. These findings imply that further research endeavors are requisite to gain a more profound understanding of the intricate relationship between the BE and the mental well-being of the aged population.

This comprehensive review meticulously amalgamates and underscores the significance of BE elements, elucidating the discoveries pertaining to the interplay between diverse BE components and MH. Our findings indicate that the proportion of green spaces within the BE, accessibility to amenities, the comparative magnitudes of building densities, and the proximity of destinations may all hold crucial importance for mental well-being. Specifically, the percentage of urban green spaces and the level of ease in reaching amenities exhibit notable correlations that can positively impact the MH of older individuals. In a longitudinal study of older adults living in American communities, the association between street distance and urbanization was investigated, the study demonstrated a negative correlation between street distance and symptoms of depression and anxiety (−0.02; 95% CI: −0.03, 0.00) (30). The percentage of green space, particularly tree canopy and forest areas, was significantly associated with better self-rated general health and a reduced likelihood of serious psychological distress (34). A study conducted in Dalian, China, compared the associations of street view, land use, and satellite-derived green space measures with MH among the older adults. The results indicated that the Normalized Difference Vegetation Index (NDVI), vegetation coverage, and park coverage were positively correlated with MH in the older adult population (8, 9). Numerous studies have revealed a significant correlation between low-density BE in developed countries and MH among the older adults. One study utilized Baidu’s Points of Interest (POI) data and data from 20 communities in Guangzhou, China, to explore the relationship between the BE and MH in the older adults. The findings indicated that facility accessibility and proximity to parks were significantly positively correlated with MH and the number of public transportation stations for the older adults, while distance to these stations was significantly negatively correlated with their MH (25, 26). In an investigation of MH among the older adults in Hong Kong communities, the remote mediating pathways from objective BE to MH and subjective well-being were scrutinized through perceived BE and community consciousness. The findings revealed an inverted U-shaped correlation between street connectivity and MH, with park green spaces exhibiting a protective effect on both MH and subjective well-being (48).

Older individuals afflicted with MH issues, including depression and anxiety, notwithstanding the presence of diverse medical facilities and pharmacological interventions, frequently encounter an economic hurdle in terms of affordability. Psychiatric medications, while effective in treating these conditions, can be notably costly, rendering them inaccessible for long-term treatment by certain economically disadvantaged groups of older people (70). It is worth noting that the socio-economic background of seniors plays a pivotal role in their MH, particularly for women (12–14). Utilizing longitudinal data from the British Biological Sample Bank, has discovered that middle-aged and older adults who were persistently exposed to green spaces encompassing a 1,000-meter buffer zone around urban areas exhibited a notably reduced risk of psychosis, specifically a 31.8 per cent decrease (40). Most research findings indicate that various characteristics of the BE are well-known to foster mental well-being. Our results may serve as a valuable guide for future research exploring the impact of urban design elements on MH.

### 4.2 Gaps identified in this review

Despite the expanding corpus of research, significant gaps remain. Geographically, studies are heavily concentrated in Asia, with limited representation from regions such as South America. This imbalance restricts the generalizability of findings across diverse cultural and environmental contexts. Methodologically, most studies employ cross-sectional designs, which fail to account for dynamic exposure to different BE elements over time. Longitudinal studies are needed to address this limitation and establish causality. Furthermore, the issue of residential self-selection—where individuals choose living environments based on personal preferences—remains insufficiently addressed. Older adults, often constrained by socio-economic factors, may have limited choices in residential location, influencing study outcomes. Larger sample sizes and more robust study designs are essential to overcome these limitations and yield actionable insights.

To delve deeper into the intricacies of MH in the older adult population and effectively propel its promotion, it is imperative to refine and optimize various facets, including research frameworks, data gathering methods, and the scope of investigation. In the realm of research design, current investigations often rely on cross-sectional approaches that hinder the revelation of causal relationships between behavioral factors and MH in this demographic. Moving forward, there is a need to upscale efforts in executing longitudinal studies and large-scale trials. By employing long-term tracking and meticulous control over confounding variables—such as self-selected residential preferences—the causal mechanisms between MH dynamics and behavioral patterns can be elucidated. Regarding data collection, existing studies often rely on static location-based measures to assess the MH of the older adults, which often miss the mark in capturing real-world environmental exposure effects. Advances in technology, particularly the integration of mobile devices and wearable sensors (77), offer the potential to dynamically monitor the activity patterns and psychological states of this population in diverse environments. By merging this real-time data with environmental factors, we can capture the true impact of these factors on MH. In the context of research scope, it is evident that there exists a notable geographical skew in current studies. A substantial 79% of the research is focused in Asia, leaving South America and Africa relatively unexplored, thereby impeding the universality of the research findings. Moving forward, studies must embrace a more expansive geographical coverage, incorporating samples from diverse cultural, economic, and environmental backgrounds. By engaging in cross-regional comparative analyses, we can discern the similarities and disparities in the impact of behavioral factors on MH in the older adult population. This approach will pave the way for formulating more targeted and widely applicable intervention strategies, ultimately propelling research on MH in the older adults from descriptive statuses to precise interventions and effective treatments.

### 4.3 Strengths and limitations

This systematic review presents a significant and perceptive understanding of the elaborate and nuanced relationship between the components of the BE and the MH of the aging population. By categorizing six key domains, it provides a framework for future research and urban planning. The widespread use of GDS-15 highlights its utility as a standardized tool for assessing MH in this demographic. However, in the meticulous literature retrieval phase of this article, we have carefully chosen only two databases, Web of Science and PubMed, as our primary sources. While there may be inherent coverage blind spots in comparison to the vast array of academic databases spread across the globe, these two databases are undeniably authoritative in their collection of academic resources. As a result, the research findings pertaining to MH and BE in the older adult population, as well as the reviews conclusions, can be confidently relied upon.

In the analysis of BE elements, the present study has endeavored to scrutinize six key dimensions: density, design, diversity, facility accessibility, road distance, and blue-green space. However, the in-depth exploration of BE diversity and urban design esthetics remains insufficient. It is crucial to recognize that BE diversity encompasses not only the abundance of functional facilities but also variations in architectural styles, landscape types, and other pertinent aspects (65). Moreover, urban design esthetics encompasses elements such as environmental visual beauty and spatial layout coordination. These factors play a pivotal role in influencing the MH of the older adults through their psychological feelings and behavioral activities (78). Future research endeavors should strive to refine the evaluation indicators for BE, delving into the mechanisms of how these often over looked elements can impact the mental well-being of the older adults. A more nuanced and comprehensive exploration is essential to fully understand the complex interplay between these elements and the psychological and behavioral aspects of the older adult population.

Most studies employed cross-sectional design, limited their ability to establish causality. Prospective cohort studies or quasi-natural experimental designs are essential for uncovering causal relationships. Due to the significant changes in the MH status of the older adults and their needs for the BE with age, longitudinal studies are helpful in tracking the long-term impact of environmental factors on their MH. Additionally, the influence of residential self-selection bias—the preference for specific living environments—may confounder results. Future research should control for this bias by measuring residential preferences and incorporating them as covariates. Another limitation is the small sample sizes in many studies, which constrains the generalizability of findings. Expanding research to a national scale would enhance the reliability and applicability of conclusions.

## 5 Conclusion

This review underscores the crucial role of BE elements in shaping the MH of older adults. Key elements, including green spaces, accessibility, and proximity to amenities, demonstrate strong correlations with mental well-being. However, research on density, diversity, and design remains sparse, and conclusions across studies lack consistency. To bridge these lacunae, forthcoming investigations ought to employ quasi-experimental designs and meta-analyses for the purpose of fortifying the evidential basis regarding the influence of the BE on MH. We have established a quantifiable evaluation framework for BE elements, addressing the limitations of previous studies that relied on subjective perception measurements, resolving the fragmentation observed in early evaluations, and developing a gradient quantification index system for BE elements suitable for the older adults, providing a more reliable and objective basis for understanding the impact of BE on MH in the older adults.

Drawing from our research findings, we have formulated four key intervention measures for policymakers and urban planners: Amplifying Green Spaces: The government must explicitly stipulate in urban planning that a set proportion of green space must be reserved during new community development or renovation. For instance, establishing a mandate for a minimum of 200 square meters of green space per 1,000 square meters of residential area. Additionally, a dedicated fund should be established to finance the creation and maintenance of urban green spaces, encompassing plant purchases, gardening staff, and recreational facilities. Enhancing Transportation Accessibility: This involves expanding public transportation routes and stops, particularly in areas with a high concentration of older adult residents. Simultaneously, improving the amenities within public transportation vehicles, such as the installation of more handrails and seats, to facilitate smooth boarding and alighting for the older adults.

Thirdly, we propose the rational distribution of public service facilities to reduce travel time and distance for the older adults. Essential services like hospitals, supermarkets, and banks should be situated close to residential areas, making it easier for the older adults to access them within a 15-min walk. Additionally, we encourage the development of community-based small businesses, such as convenience stores and pharmacies, to meet daily needs and minimize travel burdens. Fourthly, we emphasize the importance of conducting thorough research on density, diversity, and design elements. The government should allocate special research funds to explore the intricate relationship between these factors and the MH of the older adults. In summary, by identifying actionable built environment features, this review provides a foundation for creating sustainable environments that support the mental health of the global aging population.

## Data Availability

The original contributions presented in the study are included in the article/Supplementary material, further inquiries can be directed to the corresponding author.
