# Supplementary material for: A systematic review on the associations between built environment and mental health among older people
Source: Front Public Health. 2025 Jul 2;13:1584466. doi: 10.3389/fpubh.2025.1584466 (PMC12265954; doi:10.3389/fpubh.2025.1584466)
Supplement: Supplementary file 1 [file Table_1.docx]

| Supplementary Data1 **Table S1 Results of article search** | | | |
| --- | --- | --- | --- |
| **number** | **Keyword combination** | **Web of science** | **Pebmed** |
| 1 | built environment，older adults，mental health | 231 | 15 |
| 2 | built environment，older adults，psychological well-being | 29 | 5 |
| 3 | built environment，older adults，emotional well-being | 6 | 6 |
| 4 | built environment，elderly，mental health | 62 | 13 |
| 5 | built environment，elderly，psychological well-being | 8 | 4 |
| 6 | built environment，elderly，emotional well-being | 1 | 6 |
| 7 | built environment，geriatric population，mental health | 5 | 0 |
| 8 | built environment，senior citizens，mental health | 5 | 1 |
| 9 | built environment，geriatric population，psychological well-being | 0 | 0 |
| 10 | built environment，geriatric population，emotional well-being | 0 | 0 |
| 11 | built environment，senior citizens，psychological well-being | 0 | 0 |
| 12 | built environment，senior citizens，emotional well-being | 0 | 0 |
| 13 | urban environment，older adults，mental health | 275 | 39 |
| 14 | urban environment，older adults，psychological well-being | 36 | 11 |
| 15 | urban environment，older adults，emotional well-being | 10 | 11 |
| 16 | urban environment，elderly，mental health | 99 | 42 |
| 17 | urban environment，elderly，psychological well-being | 17 | 10 |
| 18 | urban environment，elderly，emotional well-being | 5 | 12 |
| 19 | urban environment，geriatric population，mental health | 8 | 6 |
| 20 | urban environment，senior citizens，mental health | 1 | 1 |
| 21 | urban environment，geriatric population，psychological well-being | 0 | 0 |
| 22 | urban environment，geriatric population，emotional well-being | 0 | 1 |
| 23 | urban environment，senior citizens，psychological well-being | 0 | 0 |
| 24 | urban environment，senior citizens，emotional well-being | 0 | 0 |
| 25 | community environment，older adults，mental health | 275 | 169 |
| 26 | community environment，older adults，psychological well-being | 65 | 53 |
| 27 | community environment，older adults，emotional well-being | 22 | 85 |
| 28 | community environment，elderly，mental health | 143 | 168 |
| 29 | community environment，elderly，psychological well-being | 15 | 50 |
| 30 | community environment，elderly，emotional well-being | 0 | 81 |
| 31 | community environment，geriatric population，mental health | 16 | 16 |
| 32 | community environment，geriatric population，psychological well-being | 1 | 5 |
| 33 | community environment，geriatric population，emotional well-being | 0 | 17 |
| 34 | community environment，senior citizens，mental health | 5 | 0 |
| 35 | community environment，senior citizens，psychological well-being | 2 | 0 |
| 36 | community environment，senior citizens，emotional well-being | 0 | 0 |
| 37 | community environment，senior citizens，emotional well-being | 151 | 19 |
| 38 | residential environment，older adults，psychological well-being | 15 | 8 |
| 39 | residential environment，older adults，emotional well-being | 8 | 10 |
| 40 | residential environment，elderly，mental health | 56 | 18 |
| 41 | residential environment，elderly，psychological well-being | 11 | 8 |
| 42 | residential environment，elderly，emotional well-being | 0 | 8 |
| 43 | residential environment，geriatric population，mental health | 5 | 1 |
| 44 | residential environment，geriatric population，psychological well-being | 0 | 0 |
| 45 | residential environment，geriatric population，emotional well-being | 0 | 1 |
| 46 | residential environment，senior citizens，mental health | 0 | 0 |
| 47 | residential environment，senior citizens，psychological well-being | 0 | 0 |
| 48 | residential environment，senior citizens，emotional well-being | 0 | 0 |
| 49 | green space，older adults，mental health | 121 | 12 |
| 50 | green space，older adults，psychological well-being | 12 | 1 |
| 51 | green space，older adults，emotional well-being | 5 | 1 |
| 52 | green space，elderly，mental health | 65 | 12 |
| 53 | green space，elderly，psychological well-being | 12 | 1 |
| 54 | green space，elderly，emotional well-being | 1 | 2 |
| 55 | green space，geriatric population，mental health | 4 | 0 |
| 56 | green space，geriatric population，psychological well-being | 0 | 0 |
| 57 | green space，geriatric population，emotional well-being | 0 | 0 |
| 58 | green space，senior citizens，mental health | 4 | 1 |
| 59 | green space，senior citizens，psychological well-being | 0 | 0 |
| 60 | green space，senior citizens，emotional well-being | 0 | 0 |
| 61 | green area，older adults，mental health | 74 | 14 |
| 62 | green area，older adults，psychological well-being | 6 | 0 |
| 63 | green area，older adults，emotional well-being | 2 | 0 |
| 64 | green area，elderly，mental health | 28 | 14 |
| 65 | green area，elderly，psychological well-being | 5 | 3 |
| 66 | green area，elderly，emotional well-being | 0 | 0 |
| 67 | green area，geriatric population，mental health | 3 | 0 |
| 68 | green area，geriatric population，psychological well-being | 0 | 0 |
| 69 | green area，geriatric population，emotional well-being | 0 | 0 |
| 70 | green area，senior citizens，mental health | 5 | 2 |
| 71 | green area，senior citizens，psychological well-being | 0 | 0 |
| 72 | green area，senior citizens，emotional well-being | 0 | 0 |
